# Endoscopic Ultrasound Guided Biliary Drainage in Malignant Distal Biliary Obstruction

**DOI:** 10.3390/cancers15020490

**Published:** 2023-01-12

**Authors:** Danilo Paduano, Antonio Facciorusso, Alessandro De Marco, Andrew Ofosu, Francesco Auriemma, Federica Calabrese, Ilaria Tarantino, Gianluca Franchellucci, Andrea Lisotti, Pietro Fusaroli, Alessandro Repici, Benedetto Mangiavillano

**Affiliations:** 1 Gastrointestinal Endoscopy Unit, Humanitas Mater Domini, 21053 Castellanza, Italy; 2 Gastroenterology Unit, Department of Surgical and Medical Sciences, University of Foggia, 71122 Foggia, Italy; 3 Gastroenterology and Digestive Endoscopy Unit, Department of Medicine, The Pancreas Institute, University Hospital of Verona, 37100 Verona, Italy; 4 Endoscopy Unit, Humanitas Clinical and Research Center IRCCS, 20089 Rozzano, Italy; 5 Division of Gastroenterology and Hepatology, University of Cincinnati, Cincinnati, OH 45201, USA; 6 Endoscopy Service, Department of Diagnostic and Therapeutic Services, IRCCS-ISMETT, 90100 Palermo, Italy; 7 Gastroenterology Unit, Hospital of Imola, University of Bologna, 40121 Bologna, Italy; 8 Department of Biomedical Sciences, Humanitas University, 20089 Rozzano, Italy

**Keywords:** MBO, EUS, LAMS, EUS-BD, cancer, stent

## Abstract

**Simple Summary:**

EUS-guided biliary drainage (EUS-BD) has proven effective in the palliation of malignant biliary obstructions. There are several methods for obtaining EUS-guided biliary drainage when endoscopic retrograde cholangiopancreatography (ERCP) fails. To date, EUS-BD, along with the well-established role of percutaneous transhepatic biliary drainage (PTBD) and surgical hepaticojejunostomy, has been demonstrated as a viable palliative treatment option in these patients. There are no specific guidelines with regard to the optimal drainage method. In this review, we compare all these techniques, demonstrating that EUS-BD in expert hands represents a minimally invasive and effective method in the palliative treatment of patients with malignant biliary obstruction.

**Abstract:**

Malignant biliary obstruction (MBO) is a challenging medical problem that often negatively impacts the patient’s quality of life (QoL), postoperative complications, and survival rates. Endoscopic approaches to biliary drainage are generally performed by ERCP or, in selected cases, with a percutaneous transhepatic biliary drainage (PTBD). Recent advances in therapeutic endoscopic ultrasound (EUS) allow drainage where previous methods have failed. EUS has evolved from a purely diagnostic technique to one that allows a therapeutic approach in the event of ERCP failure in distal MBO. Moreover, the introduction of dedicated accessories and prostheses for EUS-guided transmural biliary drainage (EUS-BD) made these procedures more successful with regard to technical success, clinical outcomes and reduction of adverse events (AEs). Finally, lumen-apposing metal stents (LAMS) have improved the therapeutic role of the EUS. Subsequently, the electrocautery enhanced tip of the LAMS (EC-LAMS) allows a direct access of the delivery system to the target lumen, thereby simplifying and reducing the EUS-BD procedure time. EUS-BD using LAMS and EC-LAMS has proven effective and safe with a low rate of AEs. This review aims to evaluate biliary drainage techniques in malignant obstruction, focusing on the role of EUS biliary drainage by LAMS.

## 1. Introduction

Malignant biliary obstruction (MBO) is secondary to different malignancies of the pancreaticobiliary system, including pancreatic ductal neoplasia, cholangiocarcinoma, ampullary or duodenal neoplasia, gallbladder neoplasia and, more rarely lymphoma or compressive metastatic lymph nodes [1]. Pancreatic ductal adenocarcinoma and cholangiocarcinoma are the most common causes of MBO and, at the time of diagnosis, are generally unresectable [2]. Bile duct occlusion leading to jaundice is commonly the initial sign of the disease and is frequently accompanied by debilitating symptoms and pruritus. Palliative endoscopic biliary decompression has become an essential method in the management of these patients [3,4]. Nonetheless, preoperative biliary drainage is not indicated for surgical candidates due to the high risk of infectious complications, although some situations may be beneficial [5]. In patients with MBO, therapeutic endoscopy ERCP and endoscopic ultrasonography (EUS) play a pivotal role in biliary decompression. In addition to jaundice palliation, these procedures offer accurate diagnosis through cytology brushing during ERCP [6] or fine needle aspiration (FNA) or fine needle biopsy (FNB), if EUS is performed [7]. Bilirubin value normalization is, moreover, mandatory before starting a neoadjuvant or palliative chemotherapy in advanced and unresectable diseases [8]. In MBO, endoscopic treatment by ERCP with stent placement generally leads to an immediate clinical response [9]. When ERCP fails, therapeutic EUS still remains the best option in patients with MBO to achieve a jaundice palliation [10]. In this review, we will focus on EUS-guided biliary drainage (EUS-BD) in malignant distal biliary obstruction.

## 2. Endoscopic Retrograde Cholangiopancreatography (ERCP), Percutaneous Transhepatic Biliary Drainage (PTBD) and EUS-Guided Biliary Drainage (EUS-BD) in MBO

For non-surgical candidate patients requiring biliary drainage for MBO, there has been much debate over the optimal method. Endoscopic drainage has virtually replaced surgery for biliary obstruction, irrespective of the possible cause. Traditionally, ERCP has been favored over surgery [11] and percutaneous transhepatic biliary drainage (PTBD) in cases with malignant biliary obstruction [12], as it has proven to be less expensive, and associated with low morbidity, and a better quality of life if compared to PTBD [6,13]. To date, in non-surgical candidate patients with MBO, ERCP plus common bile duct (CBD) stenting is considered the therapeutic option of choice [14]. However, in some cases, ERCP is not feasible, such as when the papilla is inaccessible due to altered anatomy or duodenal obstruction, or when the CBD cannot be cannulated due to tumor infiltration of the papilla.

The success of ERCP depends on several factors related to the patient’s anatomy, including post-surgical alteration, intra-diverticular papilla, neoplastic process leading to narrow malignant biliary stenosis, infiltrated ampullary area, inaccessibility to the ampullary region due to duodenal infiltration, and endoscopist’s experience. In some of these cases, and when the papillary area is accessible, advanced cannulation techniques (such as precut fistulotomy with needle knife, or septotomy with the standard papillotome) may be required, but with a high incidence of adverse events (AEs) [15]. When ERCP fails, biliary decompression should be achieved through PTBD, which is highly effective but associated with significant morbidity, recurrent occlusion and dislocation of the external catheter, and AEs such as cholangitis, bile leak or hemobilia with a significant AEs rate that can reach 26% [16,17]. Moreover, the presence of an external drainage catheter, as in the case of PTBD, can cause pain at the puncture site, along with physical discomfort related to external drainage, decreasing the quality of life (QoL) of these patients [16]. The advent of therapeutic EUS has contributed to the expansion of endoscopic biliary drainage techniques [2]. Therapeutic biliary EUS was first described by Giovannini et al. in 2001 [18], using accessories borrowed from the ERCP armamentarium [19,20,21,22]. The introduction of dedicated accessories for EUS-guided transmural biliary drainage (EUS-BD) has provided a novel and effective alternative method for the palliation of jaundice, and especially with the advent of lumen-apposing metal stent (LAMS).

Multiple published studies have demonstrated that EUS-BD with LAMS is a safe and effective procedure for patients with an unsuccessful ERCP attempt [23,24,25,26]. LAMSs allow side-by-side juxtaposition of two hollow organs creating an anastomosis, preventing stent migration. The development of the electrocautery-enhanced (EC) LAMS tip facilitates direct access to the biliary system, simplifying the procedure [27].

The disadvantages of the device include greater rigidity and limited insertion depth of the delivery catheter, retrograde sliding of the LAMS during deployment of the distal flange (only described for Hot-Axios, Boston Scientific, Natick Mass, MA, USA) and high costs [28,29].

One of the first reports about this issue was published by Kunda et al. He showed, in a retrospective series of 57 patients who underwent EUS-BD for MBO by LAMS after unsuccessful ERCP, a technical success of 98.2% with an overall clinical success of 94.7%. Reported AEs were 7% due to two duodenal perforations, one bleeding and one transient cholangitis. A total of 9.3% of the patients with clinical success required re-intervention, due to stent migration in one case and a sump syndrome with transient increase in serum bilirubin concentrations in the remaining patients [30]. A comprehensive literature review by Jain et al. demonstrated how, in expert hands, EUS-BD by LAMS is an efficacious and safe option for patients with distal MBO, not amenable to ERCP.

A high degree of technical success was observed in patients with a limited dilation of the extrahepatic biliary tree (less than 1 cm) and altered gastrointestinal anatomy (Roux-en-Y, Whipple, or Billroth II). Clinical success was reported in 98.9% of patients. The incidence of AEs was 12,1% including perforation (2.2%), bleeding (1.1%), obstruction (7.7%), and migration (1.1%).

All the complications were endoscopically treated, including the debridement of tumor or food, the placement of a plastic pigtail stent through the LAMS, and stent replacement. No procedure-related mortality was observed [31].

In addition, the EUS-BD by LAMS is reported as a primary alternative approach to ERCP in MBO replacing the PTBD, with high technical and clinical success [28]. EUS-BD is a salvage procedure for patients with inaccessible papilla or failed ERCP, and it has been demonstrated to be superior to PTBD [32]. A meta-analysis from Sharaiha et al. involving nine studies with 483 patients found no difference in technical success between EUS-BD and PTBD (OR, 1.78; 95% CI, 0.69–4.59; I2 = 22%), but EUS-BD was associated with better clinical success (OR, 0.45; 95% CI, 0.23–0.89; I2 = 0%), fewer post-procedure adverse events (OR, 0.23; 95% CI, 0.12–0.47; I2 = 57%), and lower rate of reintervention (OR, 0.13; 95% CI, 0.07–0.24; I2 = 0%) [33]. The multicenter trial conducted by Lee et al. confirmed these data and showed a procedure-related adverse of 8.8% in the EUS-BD group vs. 31.2% in the PTBD group (*p* = 0.022); the mean frequency of unscheduled re-intervention was 0.34 in the EUS-BD group vs. 0.93 in the PTBD group (*p* = 0.02), while the QOL was comparable between groups [34].

## 3. Types of LAMS Currently Available

Lumen apposing metal stents (LAMSs) are self-expanding, fully covered devices, capable of forming a stable anastomosis between adjacent organs and cavities. LAMS is a new type of fully covered self-expandable metal stent (FCSEMS) mounted on a delivery system with an electrocautery distal tip, with a short body and large flanges [35,36,37,38,39].

It has a “yo-yo” shape that enables the juxtaposition of two hollow organs to create an anastomosis and prevent stent migration.

LAMSs are made up of nitinol and are covered with a silicon layer, which helps prevent tissue ingrowth and facilitates easy removal. These characteristics allow for more minimally invasive procedures with the potential for higher rates of success and fewer adverse events (AEs) [40].

With the subsequent application of the electrocautery-enhanced tip, the EC-LAMS has enabled a “free-hand”, “single-step” and “exchange-free” procedure without using further devices. EC-LAMS does not require additional accessories such as needles, guidewires, or dilator devices, allowing for a reduction in complexity and risk for adverse events. The electrocautery-enhanced tip makes direct organ access possible.

The surface area of the electrode is very small, corresponding to only 3% of a 10 Fr cystotome, resulting in a high current density and precise, controlled access to the target organ [41]. The electrocautery-enhanced delivery system simplifies the execution of the EUS-guided procedures as EUS-guided choledochoduodenostomy (EUS-CDS) or gallbladder drainage (EUS-GBD), by reducing the technical steps and operative time.

**Figure 1:**
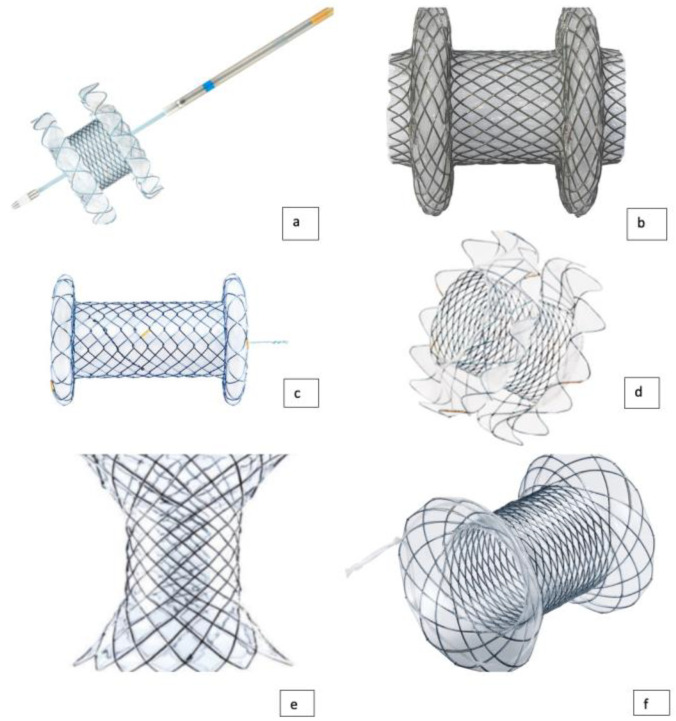
(**a**) Hot-Spaxus stent (TaeWoong Medical); (**b**) Axios stent (Boston Scientific); (**c**) Hanarostent Plumber stent (M.I. Tech); (**d**) Spaxus stent (TaeWoong Medical); (**e**) Aixstent PPS (Leufen Medical); (**f**) Nagi stent (TaeWoong Medical).

Numerous lumen-apposing metal stents (LAMS) have been designed for transluminal applications. Nowadays, different types of LAMS are available on the market: Axios (Boston Scientific), Spaxus and NAGI (Taewoong Medical Co), Aixstent PPS (Leufen Medical) and Hanarostent (M.I. Tech). Currently, only the Hot-Axios stent is available in the United States for human use, while the other LAMS, such as the Hot-Spaxus stent (Taewoong Medical Co, Gimpo, Korea) are not available worldwide [42] (Figure 1). The Hot-Spaxus was recently developed with the incorporation of an electrocautery tip as an evolution of the previous Niti-S Spaxus [43]. Hot-Axios is shaped like a dumbbell or saddle shape with terminal flanges. The inner diameter (ID) could be 6, 8, 10, 15 and 20 mm with a body length from 10 to 15 mm.

Similar in structure to the Niti-S Spaxus, it is actually distributed in three different inner diameters: 8, 10 and 16 mm with, respectively, flange dimensions of 23, 25 and 31 mm. The length of the devices is 20 mm, and there is a 7 mm gap between the flanges.

The Nagi stent is designed with flared ends to reduce stent migration, and is marketed in four different inner diameters: 10, 12, 14, or 16 mm and lengths of 10, 20, and 30 mm. Axistent, which is available in Europe but not yet available in the US, is manufactured with an inner dimeter of 10 and 15, a total length of 30 mm, with flanges of 25 mm wide.

Finally, the Hanarostent Plumber is available in 10, 12,14 or 16 mm inner diameter with usable lengths of 10 or 30 mm. The outer flange diameter ranges from 22 to 28 mm. All the exposed devices are made of nitinol and silicon and all of them are placed through the scope mechanism [19,43,44].

## 4. EUS-Guided Biliary Drainage Techniques

The advent of interventional EUS dates back to 20 years ago, and the introduction of dedicated accessories and prostheses for transmural EUS-BD has provided a novel and effective alternative approach for jaundice palliation in patients with MBO [8].

**Figure 2:**
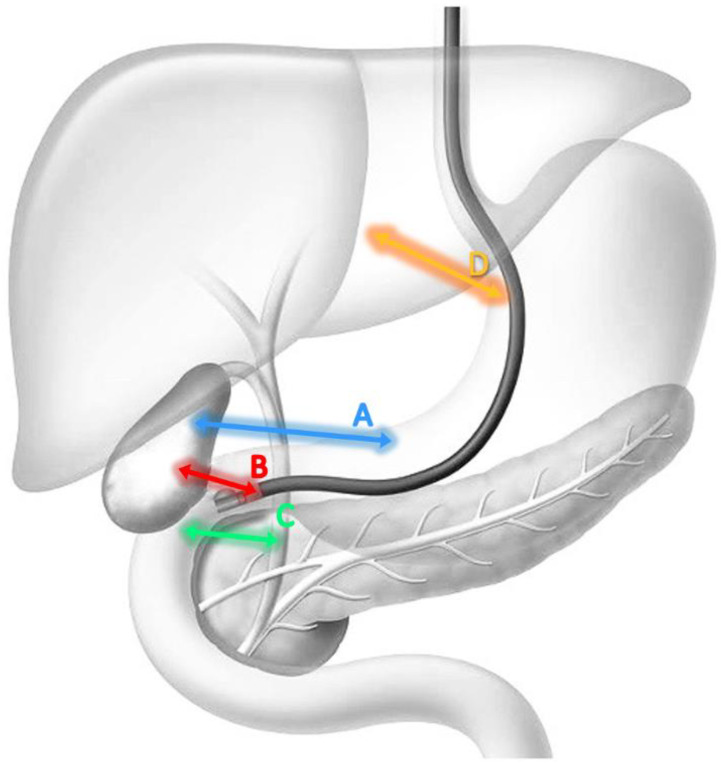
This figure describes the EUS-guided drainage techniques: (**A**) Cholecystogastrostomy; (**B**) cholecystoduodenostomy; (**C**) choledochoduodenostomy; (**D**) hepaticogastrostomy.

The EUS-BD for distal MBO can be performed by three different techniques:

(1) cholecystogastrostomy (Figure 2A)
(2) cholecystoduodenostomy (Figure 2B)
(3) choledochoduodenostomy (Figure 2C)

This technique was firstly described by Giovannini in 2001, using a 5 Fr needle-knife for CBD puncture with subsequent 10 Fr plastic stent placement over a biliary guidewire [18]. The drainage technique has undergone changes over the years, and thanks to the advent of the LAMS this has become simpler and safer. Initially, the drainage was performed using a multistep technique [45,46,47] (MST) schematized as follows:

- access the target organ by using a 19 G EUS FNA needle;
- aspiration of 2–3 mL of bile to confirm the position of the needle tip;
- contrast injection from the 19 G FNA needle (2–3 mL) for the anatomy study;
- 0.035”, 0.025” or 0.032” guidewire insertion;
- FNA needle exchange with a 6 Fr cystotome or a 4 mm balloon to dilate the tract;
- stent placement.

With the advent of EUS guided biliary drainage, plastic stents were initially used. Frequent complications, including early occlusion necessitating stent replacement and migration, represent a risk associated with the use of plastic stents [48]. The insertion of the dilating device, which carries a risk of non-penetration into the target organ, is one of the most crucial steps of the procedure. This could be a result of the guidewire being excessively coiled, resulting in a tangential direction of the dilating device and subsequent difficult access through the walls of the two organs.

The introduction of the EC-LAMS has resolved these issues. In addition, it has limited the use of fluoroscopic guidance during their placement. Finally, the intrachannel stent release (ICSR) technique to perform stent placement under complete EUS control, without the use of fluoroscopic guidance has, additionally, shortened the procedure time [41]. The target organ is directly punctured using a cautery-enhanced device, with free-hand technique. Then, the LAMS is pushed inside the target organ and the distal flange is deployed under EUS guidance. A subsequent slight retraction of the delivery system creates an apposition with the target organ’s wall, and the proximal flange is deployed inside the operative channel of the echoendoscope and pushed outside under endoscopic control (ICRS). The proximal flange of the LAMS can be also released directly under endoscopic surveillance without its opening inside the operative channel; this requires a small echoendoscope retraction to create space between the stomach/duodenum and the tip of the endoscope. Unlike the traditional technique, in which the proximal flange is deployed under endoscopic control, the ICRS permits a more stable position [41]. The use of fluoroscopic guidance is optional, but an X-ray setting is strongly recommended if AEs occurred and a salvage procedure should be performed.

For detailed anatomical information prior the LAMS placement, a computed tomography (CT) or magnetic resonance imaging (MRI) of biliary anatomy is required, and the case should be discussed in a preprocedural interdisciplinary approach involving the endoscopist, interventional radiologist, surgeon and oncologist.

Endoscopic examination of the stomach and duodenum is also essential for ruling out organs’ lesions that could affect the LAMS procedure. During biliary drainage (both GB and CBD) it is also necessary to examine the puncture site, particularly for the evaluation of any interposed vessels by color Doppler. If the duodenal approach is possible, the echoendoscope is inserted into the duodenal bulb and the common bile duct or gallbladder is identified; the long position is typically preferable during the LAMS placement because of more stability than the straight scope [45]. However, in the long position, insertion of the LAMS Delivery System is more difficult due to greater resistance in the curved endoscope channel than it is during the short position. If there is difficulty in releasing the delivery system, it is recommended to shorten the endoscope to facilitate the exit of the device from the canal and then resume the correct position to perform the procedure.

The color Doppler study is performed prior to the device entrance in the targeted organs to exclude interposed vessels. In the case of choledochoduodenostomy, careful monitoring of the sign of the double mucosa is important in order to avoid complications such as bleeding and perforation [49]. If the endosonographer prefers, a guidewire is advanced after puncturing the common bile duct or gallbladder; otherwise, the access should be performed using a freehand technique.

Under EUS guidance, the stent delivery system is then inserted. The use of LAMS facilitates the adhesion between the target lesion and the gastrointestinal lumen with a high apposition force; in addition, the design of the stent, with larger ends, prevents stent migration.

The use of EC-LAMS permits the insertion of the stent delivery system without dilation of the fistula and with the reduction of the adverse events associated with the loss of bile, as well as a reduction of the duration of the procedure and the duration of fluoroscopic exposure [50].

In case of gallbladder drainage from the stomach, the access is typically through the gallbladder body, whereas, if the access is through the duodenum, the GB is frequently punctured in the neck.

EUS-gallbladder drainage though the stomach is easier than the CBD or GB drainage from the duodenum because there is more working space within the gastric cavity to locate the optimal access point and to release the proximal flange [51]. There are few studies comparing transgastric with transduodenal EUS-GBD and no statistically significant differences have been found [52]. In conclusion, in the presence of distal MBO in surgically unfit patients, the drainage route is determined solely by the patient’s anatomy (Table 1).

When the papilla is inaccessible due to duodenal strictures or ulcers caused by malignancy or surgically altered anatomy, EUS-guided hepaticogastrostomy (EUS-HGS) represents a useful option [53]. This work intends to focus on EUS-guided biliary drainage using LAMS; however, it is useful for the reader to describe EUS-HGS as a further option. The procedure is performed with a therapeutic echoendoscope positioned in the stomach and by ultrasound identifying the II or III hepatic segment. Intrahepatic access to the dilated peripheral bile ducts is achieved by transgastric puncture, using a 19-gauge needle. After access to the biliary tree has been confirmed, a 0.025 inch guidewire is advanced into the common bile duct. A 6-Fr cystotome using monopolar current in Autocut mode is subsequently advanced through the gastric wall, into the dilated segmental duct under fluoroscopic guidance and EUS. Finally, a fully covered (8 × 10 mm or 6 × 10 mm, Wallflex Rx biliary stent, Boston Scientific, Marlborough, Massachusetts, USA) or partially covered self-expandable metal stent (SEMS) (Giobor, Taewoong Medical, Gimpo, South Korea or Hanarostent, M.I. Tech Co. Ltd., Gyeonggi-do, South Korea) is deployed, creating a hepaticogastrostomy (Figure 2D) [54]. This drainage technique can also be used in complex hilar strictures with significant invasion of hilum and right liver, when ERCP is associated with a high failure risk [55]. EUS-HGS can be used as an initial drainage method, but can also represent a rescue method after ERCP failure. In the latter case, EUS-HGSs have shown comparative efficacy to PTBD with fewer adverse events. Compared to PTBD, when performed by experts EUS-HGS is a more comfortable and physiologic procedure for patients due to internal drainage. The overall number of reoperations appears to be lower after EUS-HGS than after PTBD. However, given the complexity and the lack of a dedicated device, EUS-HGS is still a limited procedure which can be burdened by even very serious adverse events due to the anatomical proximity to the mediastinum. The success rate of EUS-HGS is comparable to ERCP and PTBD procedures. Furthermore, hepaticogastrostomy reduces the risk of acute pancreatitis associated with ERCP-related papillary irritation. Since the stents are not placed across stricture sites, the stent patency can be longer in EUS-HGS then in endoscopic retrograde biliary drainage [56]. A work by Paik WH et al. demonstrated that the technical and clinical success rates of EUS-HGS were 96% and 90%, respectively, comparable to that of EUS-guided choledocoduodenostomy (EUS-CDS). However, EUS-CDS is more widely used because extrahepatic biliary access through EUS is closer and easier. However, EUS-HGS may be preferred over EUS-CDS as an alternative to ERCP when this is not feasible, as in the case of surgically altered anatomy or duodenal obstruction. EUS-HGS appears to be a safe procedure and produces fewer procedure-related adverse events than PTBD. The overall rate of adverse events, again in the work of Paik WH et al., was 18%, mainly represented by abdominal pain, self-limiting pneumoperitoneum, bile leakage, cholangitis and bleeding. Another possible limiting consequence of the use of stents for EUS-HGS is due to their clogging by food material, which reduces their patency. Serious adverse events such as perforation, intraperitoneal migration of the stent and mediastinitis may occur in rare cases. EUS-HGS has more types of adverse events than EUS-CDS and some of them can be life threatening to the patient as the main consequence of bile leakage and occurrence of sepsis [53].

**Figure 3:**
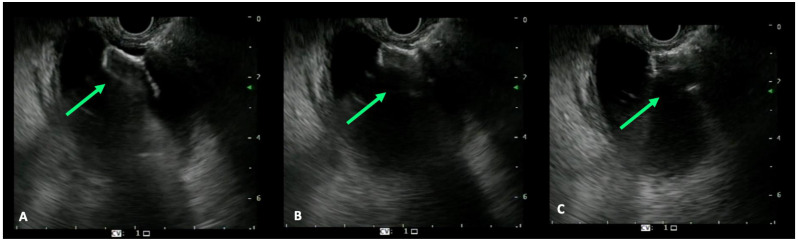
EUS view of the correct positioning of the LAMS: (**A**) completely open distal flange in the shape of a “frisbee”; (**B**) the distal flange is pulled back towards the intestinal wall; (**C**) distal flange of oval shape.

**Figure 4:**
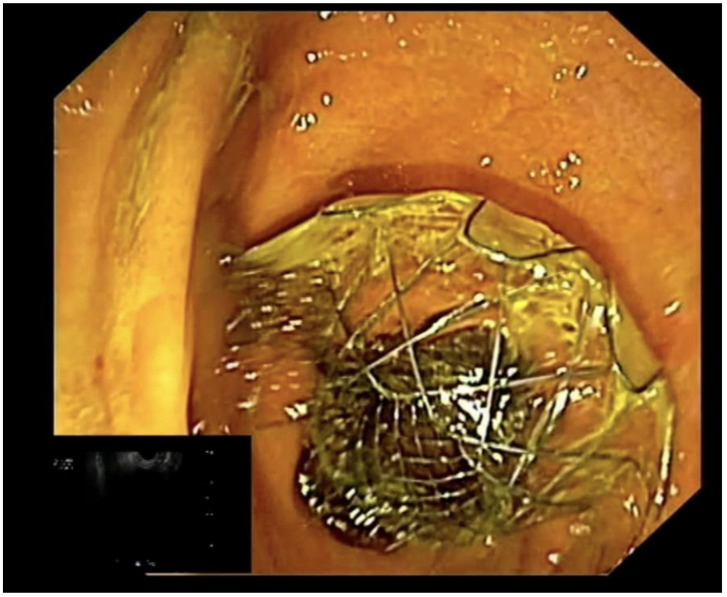
Example of correct deployment of the proximal flange.

The jaundice palliation by LAMS is also burdened by possible AEs that can occur during the procedure or in the post-procedural period. The most common AEs encountered are incorrect stent placement, migration, bleeding, perforation and infection, with an estimated rate of 16.3% [57]. EUS-guided biliary drainage should be performed under deep sedation and an X-ray setting is strongly recommended for fluoroscopic control. After identifying the target organ, the distance between the tip of the scope and the organ itself should be measured and a Doppler examination must be performed to exclude the presence of interposed vessels. The preloaded LAMS on the electrocautery delivery system should be inserted into the working channel of the echoendoscope and the luer-lock fixed must be attached to the entrance port of the working channel. Thereafter, the tip of the delivery system is advanced out of the working channel. The delivery system is connected to the electrosurgical generator (settings: pure cut mode, 80–120 Watts, 400–500 Vp; effect 5) and the penetration of the target structure is achieved by applying current while the catheter is advanced gently. After the tip is inside into the target structure, the device should be advanced and the distal flange of the stent can be deployed under EUS guidance (it should be seen fully open as a “frisbee”), maintaining the scope in a stable position (Figure 3A) [41]. The deployment of the proximal flange begins when the distal flange is completely attached to the targeted organ’s wall, after pulling back the device into the working channel until the shape of the flange changes from flat to oval (Figure 3B,C). When this change in the shape of the distal flange occurs, the proximal flange can be deployed safely within the working channel without the need for an endoscopic view. The delivery system is now gently pushed until the proximal flange completely opens outside the scope (Figure 4). The choice of the most suitable type of LAMS always remains at the discretion of the endoscopist, based on their experience and preferences. From our experience, we suggest the use of 10 mm ID LAMS for gallbladder drainage from the stomach, while using 8 mm ID LAMS from the bulb. For the drainage of the common bile duct, on the other hand, we suggest LAMS with an internal diameter of 6 or 8 mm.

## 5. Adverse Events

EUS-guided biliary drainage using LAMS is a potentially adverse event prone procedure. The operator’s experience, skill and competence in LAMS deployment help reduce the risk of adverse events [31]. Adverse events can be classified into early (periprocedural) and late (Table 2). Among the first adverse events, we include the possible complications observable during the procedure or up to one week after the procedure. These are:

-Bleeding: The risk of bleeding during the deployment of the LAMS is basically linked to lesions of blood vessels interposed or surrounding the point of penetration of the target organ. This bleeding may be self-limiting or may require life-saving procedures such as embolization for hemostasis if, for example, the gastric artery is involved [58]. A Doppler examination is essential to exclude the presence of interposed vessels before positioning the LAMS to prevent this possible and dangerous complication.

-Perforation or Pneumoperitoneum: Duodenal perforation is another possible complication observed during the procedure. In the literature, duodenal perforations have been described as caused by the tip of the echoendoscope during the evaluation of the most appropriate site to perform the drainage, or by technicians during the execution of the dilatation of the tract necessary to allow the subsequent insertion of the LAMS. In both cases, the perforation was effectively treated by endoscopic clip placement such as the OTSC (Ovesco Endoscopy GmbH, Tübingen, Germany) [30]. These complications, however, should not be attributed to the stent placement procedure. Tsuchiya et al. instead reported a case of pneumoperitoneum, which was managed conservatively with good results [59].

-Cholangitis or Fever: Patients undergoing EUS-BD via LAMS placement may develop fever or cholangitis. These complications can generally be solved by supportive therapy or, in the case of food residues inside the stent, by irrigation and mechanical removal of food residues [30,31,59].

-Stent Obstruction: Stent obstruction is due to food debris that partially or totally occludes the stent lumen. Endoscopic cleaning with the removal of food debris is necessary and effective in correcting this complication [59].

Late adverse events include:

-Stent Migration: The “yo-yo” shape of the LAMS was specifically designed to prevent migration. However, some studies report that the migration rate of LAMS is between 10 and 19% [60,61,62]. Migration can occur immediately due to improper placement of the LAMS, or weeks after stent placement, and also due to subsequent stent handling. LAMS can migrate into the target organ cavity or back into the gastrointestinal lumen. Migration into the target organ cavity could lead to the collapse of the transgastric or transduodenal fistulous tract and failure of the procedure. This complication should be managed with urgent endoscopic retrieval or subsequent surgery. The management of stent migration into the gastrointestinal lumen is primarily by direct endoscopic extraction. In transduodenal drainage, on the other hand, the intracanal release of the proximal flange from the duodenal bulb, if not performed under precise control, could be the cause of pyloric occlusion due to the complete coverage by the proximal flange released via the transpyloric route into the stomach [63].

-Late bleeding: EC-LAMS has been shown to be safe and has low bleeding risks; however, there are some case reports of delayed bleeding caused by LAMS. Delayed bleeding after LAMS placement is mainly due to an underlying coagulopathy [64,65].

-Buried stent: It is a condition that occurs when the ends of the stent are pulled in and embedded in the stomach wall. The LAMS flange can be only partially or completely (buried) incorporated by the mucosa. The specific cause of this complication is unclear; it probably occurs because the flanged edge of the stent is in close contact with the wall. Some studies report nearly 17% buried stent rate [66].

-Stent Obstruction: Late causes of stent obstruction are food debris, tumor growth, kinking, or dislodgement of the stent. In all these cases, endoscopic intervention with mechanical debridement of residual food, debridement of the tumor, removal of the LAMS with the placement of a new plastic stent through the fistula or the placement of a metal stent through the LAMS is required [59].

-Sump Syndrome: Sump syndrome is a rare long-term complication of biliary drainage using LAMS. This condition occurs when the part of the bile duct distal to the anastomosis is transformed into a poorly drained reservoir, making this so-called “well” subject to the accumulation of debris, stones and static bile, favoring bacterial proliferation. Patients with Sump syndrome tend to have recurrent abdominal pain and cholangitis. This complication can be successfully managed by transient placement of double-tailed plastic stents through the LAMS for the duration of two weeks [30].

## 6. Conclusions

Malignancies leading to distal MBO tend to be unresectable at presentation; therefore, palliative treatment is necessary. Palliative therapy should be minimally invasive and cost-effective, with the aim to improve the QoL of these patients or initiate chemotherapy. Endoscopic palliation strategies by EUS-drainage with LAMS for distal MBO require proper planning arranged after a multidisciplinary discussion or should be performed after a failed attempt of ERCP [67]. EUS-guided biliary drainage by LAMS is a less invasive approach for jaundice palliation in distal MBO when ERCP is not feasible; moreover, a recent published study attests the use of EUS-BD as the initial step in MBO [32]. LAMS is effective due to its adaptability and design, which permits the creation of a fistula between the two select organs. Unrecognized and incorrect deployment remains a serious complication with fatal evolution if not correctly recognized and managed. Rescue therapy must be promptly performed by highly skilled endoscopists, interventional radiologists and dedicated surgeons [68]. In conclusion, EUS-guided biliary drainage by LAMS is feasible in non-surgical candidates with distal MBO and should be performed by endoscopists with extensive experience in therapeutic EUS and ERCP. An early identification of AEs and appropriate management, if they occur, should reduce morbidity and prevent mortality. A multidisciplinary approach is required for determining the optimal therapeutic strategy for these patients.

The advent of interventional EUS, especially the use of LAMS in guided EUS drainage, has certainly opened new horizons in the management and treatment of MBO. In the future, it will be desirable to expect improved prostheses with, for example, simpler and safer methods of deployment, with smaller diameter and reduced body length or, again, it could be useful to have LAMS available with an anti-reflux valve in both GB and CBD drainage so to avoid possible ascending cholangitis.

## Figures and Tables

**Table 1:** Summary table based on the site of approach.

	Procedure	LAMS: Inner Diameter	Endoscope Position	Indications	Recommendations	Supplemental Comments
**Stomach**	Cholecystogastrostomy	8–10 mm	Long or short. Gallbladder visualized in EUS along the posterior wall/greater curvature of the stomach.	Malignant distal biliary obstruction with retro dilatation of common bile duct	Distended gallbladder with diameter ≥ 20 mm.	After locating the target organ, the distance between the tip of the endoscope and the organ itself should be measured. This distance must be ≤10 mm. Subsequently a Doppler examination must be performed to exclude the presence of vessels or other intervening structures.
**Duodenum**	Cholecystoduodenostomy	6–8 mm	Long from the duodenal bulb.	Malignant distal biliary obstruction with retro dilatation of common bile duct and sparing of the duodenal bulb.	Distended gallbladder with diameter ≥ 20 mm.	
	Choledochoduodenostomy	6–8 mm			Distended CBD with diameter ≥ 15 mm.	

**Table 2:** Adverse events early (periprocedural) and late.

Adverse Events	
Early (periprocedural)	- Bleeding - Perforation or Pneumoperitoneum - Cholangitis or Fever - Stent Obstruction
Late	- Stent Migration - Late bleeding - Buried stent - Stent Obstruction - Sump Syndrome

## Data Availability

Data sharing not applicable. No new data were created or analyzed in this study. Data sharing is not applicable to this article.
